# Supplementary material for: A Diet Containing Rutin Ameliorates Brain Intracellular Redox Homeostasis in a Mouse Model of Alzheimer’s Disease
Source: Int J Mol Sci. 2023 Mar 2;24(5):4863. doi: 10.3390/ijms24054863 (PMC10003355; doi:10.3390/ijms24054863)
Supplement: Supplementary file 1 [file ijms-24-04863-s001.zip › ijms-2205529-supplementary.pdf]

Supplementary methods

S1. FLUORO-JADE C STAINING

Fluoro-Jade C staining is a technique that allows the selective identification of degenerating neurons and their projections (distal dentrites, axons and axon terminals) in tissue sections regardless of the cause of the neurodegeneration.

Fluoro-Jade C is an anionic derivative of fluorescein with a molecular weight of 823 daltons and an excitation peak maximum at 485 nm (blue) and an emission peak maximum at 525 nm (green), and both properties are pH-dependent. For the labelling to be specific, it must be prepared in an acidic medium (pH 3-4) as it favours the intensity of the light emitted.

The method followed was described by Schmued et al. (2005)[1] with minor modifications.

Working solutions

- 0.1 M phosphate buffer, pH 7.4
- Formaldehyde 4% in phosphate buffer
- Basic alcohol: NaOH 1% in ethanol 80%
- Ethanol 70%
- Potassium permanganate 0.06%
- Acetic acid 0.1%
- Fluoro-Jade C and 0.0001% DAPI in 0.1% acetic acid. This solution was prepared on the spot from a 0.01% stock solution (stored at 4°C protected from light, stable for 3 months)
- Fluoromount™ Aqueous Mounting Medium for use with fluorescent dye-stained tissues

Procedure

Brain sections of 30 µm were pre-dried to be fixed in a 4% formaldehyde solution in 0.1 M phosphate buffer pH 7.4 for 10 min. After this time, they were washed twice in phosphate buffer, then placed in basic alcohol for 5 min. After this time, they were incubated in 70% ethanol for 2 min, washed with water for 2 min, and then incubated in 0.06% potassium permanganate for 10 min, in order to decrease the background of the staining, as well as to avoid loss of fluorescent signal.

After staining, the brain sections were washed with water for 1 min. They were then incubated in Fluoro-Jade C and 0.0001% DAPI solution for 10 min. After this time, they were washed with distilled water 3 times for 1 min at each change, and the excess water was removed with paper and 1 drop of Fluoromount™ Aqueous Mounting Medium was added. They were left to dry for 1 h. To prevent desiccation, the edges were sealed with nail polish. The sections were examined under the Zeiss Axioplan-2 epifluorescence microscope, with the excitation filter set at 485 nm and the emission filter set at 525 nm. To observe the blue nuclear labelling by DAPI, they were excited with UV light.

S2. EXPRESSION OF GLUTAMATE IONOTROPIC RECEPTORS

[1] Schmued LC, Stowers CC, Scallet AC, Xu L. Fluoro-Jade C results in ultrahigh resolution and contrast labelling of degenerating neurons. Brain Res 2005;21:1035(1):24-31.

^{3}H-AMPA autoradiography

For AMPA-type glutamatergic receptor labelling, the tritiated ligand ^{3}H-AMPA (Perkin Elmer, USA) was used. Prior to incubation with the ligand, the slices were pre-incubated with 100 mM potassium thiocyanate dissolved in 50 mM Tris-acetate (pH 7.2) at 4ºC. The pre-incubation was repeated 3 times for 10 min each. After preincubation, the slices were incubated with the same solution with the addition of the tritiated ligand at a concentration of 10 M for 45 min at 4°C. After labelling, the slices were washed 4 times in the incubation buffer without the tritiated ligand for 4 s, and then 2 times in a solution of glutaraldehyde in 2.5% acetone for 2 s. Then, the slices were dried in an oven at 37°C and placed in the cassettes together with a high-resolution autoradiographic film (Biomax MR, Kodak) for 10 weeks before development.

^{3}H-MK801 autoradiography

This ligand ^{3}H-MK801 (Perkin Elmer, USA) binds to NMDA-type glutamatergic receptors. For this purpose, coronal slices were pre-incubated in 50 mM Tris with 50 M glutamate, 30 M glycine and 50 M spermidine at pH 7.2 at 4ºC. After pre-incubation, the slices were immersed in a similar solution with 3.3 M ligand addition for 1 h at room temperature. After incubation, the slices were washed with the glutamate-free incubation solution 2 times for 5 min each wash and finally with distilled water at 4°C for 1 s. After completion of the protocol, the slides were dried in an oven at 37°C and exposed to high-resolution autoradiographic film (Biomax MR, Kodak) for 10 weeks before development.

^{3}H-Kainate autoradiography

The ligand ^{3}H-kainate (Perkin Elmer, USA) binds to glutamatergic kainate receptors. The slices were pre-incubated in a solution of 50 mM Tris-citrate and 10 mM calcium acetate at pH 7.1 at 4ºC. The pre-incubation was repeated 3 times for 10 min each. After this time, the slices were incubated in the same solution, this time with the radioactive ligand at a concentration of 9.4 M for 45 min at 4ºC. Once the incubation was finished, the slices were washed in the incubation buffer without ligand 3 times for 4 s at 4ºC and another 2 times with glutaraldehyde in 2.5% acetone for 2 s each. After the incubation protocol, the slides were dried in an oven at 37ºC. After drying, they were placed in a cassette together with a high-resolution autoradiographic film (Biomax MR, Kodak) for 12 weeks before development.

Supplementary data

Table S1. Reagent mix for cDNA synthesis.

Reactivos	Volume (µL)
RT buffer 10X	4
RNase inhibitor (20 U/µL)	1
10 mM dNTPs	2
Reverse Transcriptase (20 U/µL)	1

Table S2. Reagent mixture for a real-time PCR reaction.

Reaction ingredients	Volume (µL)
Quantimix easy master mix 2X	10
10 µM Primer forward	1
10 µM Primer reverse	1
10 mg/mL BSA	1
cDNA	1
PCR-grade water	6

Table S3. Primers for *Mus musculus* cDNA synthesis.

Gene	Oligonucleotide sequence (Sense 5´-3´)	Oligonucleotide sequence (Antisense 3´-5´)	T_A	pb
Human APP	ACCGCTGCTTAGTTGGTGAG	GGTGTGCCAGTGAAGATGAG	55	113
BACE1	CCACCAACCTTCGCTTGCCCA	CCAGCACACCAGCTGCTCCC	65	119
ADAM10	AGTGGAGCGAGAGGGAGGCG	CCGCCCAGGAGAGGAGCAGA	65	163
Glutathione reductase	TGTCAAAGGCGTCTATGCTG	GGCTGAAGACCACAGTAGGG	60	155
Superoxide dismutase-2	GCCTTCCCAGGATGCCGCTC	CGTCTGCTAGGCAGCGTCCG	65	136
Catalase	TCTCCGGGTGGAGACCGCTG	CCCGCTGCTCCTTCCACTGC	65	133
Glutathione peroxidase-1	TTTGGTCTCCGGTGTGCCGC	GGGCCGCCTTAGGAGTTGCC	65	169
Caspase-3	TCATTCAGGCCTGCCGGGGT	CTGGATGAACCACGACCCGTCC	65	176
Caspase-6	TCCAGGCCTGTCGGGGTAGC	GGACGCAGCATCCACCTGGG	65	107
IL-1β	AGCCTCGTGCTGTCGGACCC	TGAGGCCCAAGGCCACAGGT	65	139
IFN-γ	CCCACAGGTCCAGCGCCAAG	CCCACCCCGAATCAGCAGCG	65	111
TNF-α	CAAGGGACAAGGCTGCCCCG	GCAGGGGCTCTTGACGGCAG	66	109
GADPH	AGAAGGTGGTGAAGCAGGCATG	CGAAGGTGGAAGAGTGGGAGTT	64	111

Table S4. Conditions to perform real-time PCR.

Time and temperature			
**Initial denaturation**	**45 cycles**		
**Denaturation 10 min, 95ºC**	**Denaturation**	**Hybridisation**	**Elongation**
	95ºC, 3 s	Depending on primers T_A/15 s	72ºC, 20 s

S1. ASSESSMENT OF NEURODEGENERATION (STAINING WITH FLUORO-JADE C) AND NUCLEAR MORPHOLOGY OF HIPPOCAMPAL NEURONS (COUNTERSTAINING WITH DAPI)

Based on the amyloid cascade theory, it is established that the accumulation and aggregation of Aβ triggers neurodegenerative processes that ultimately lead to neuronal death. To assess whether Aβ accumulation actually induces cellular neurodegeneration, we used the dye Fluoro-Jade C, which stains cells in the process of cell degeneration. Specifically, coronal sections were stained with Fluoro-Jade C at the -2.54 position following the Bregma reference system (Figure S1).

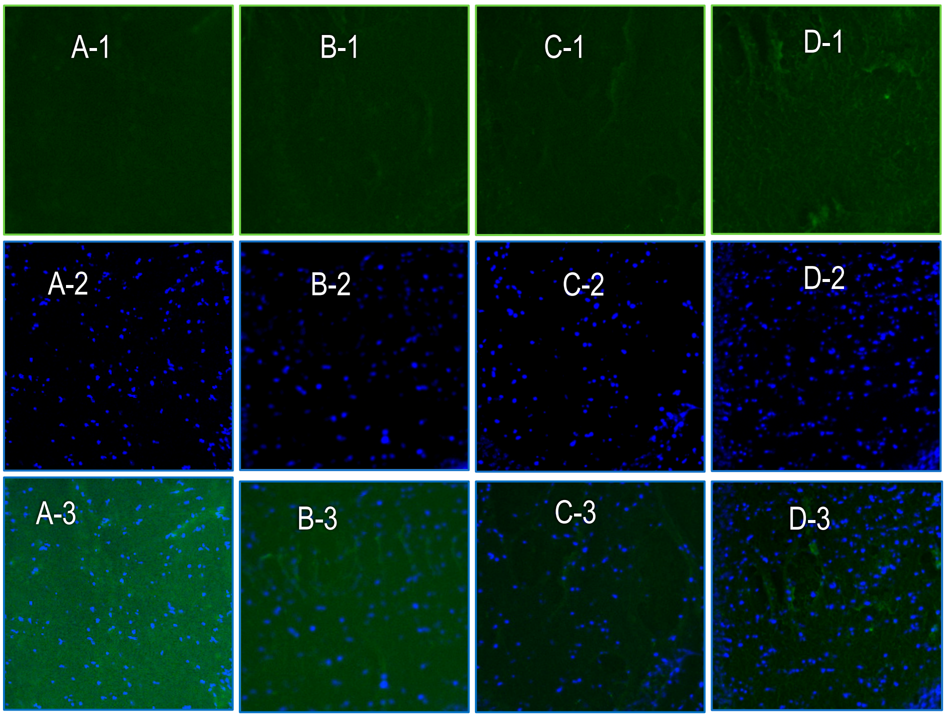

Figure S1. Observation of neurodegeneration (Fluoro-Jade C) and nuclear chromatin (DAPI) at the ventral hippocampal level. **1)** Fluoro-Jade C; **2)** DAPI; **3)** DAPI and Fluoro-Jade C overlay. **A)** WT mice; **B)** TgAPP control; **C)** TgAPP + Quercetin; **D)** TgAPP + Rutin.

No characteristic signs of neurodegeneration were observed at the age at which the transgenic TgAPP mice were evaluated, compared to WT mice, nor did treatments with quercetin and rutin for 4 weeks show any change vs TgAPP (Figure S1. A-1. B-1, C-1 and D-1).

Counterstaining with 4'-6-diamidino-2-phenylindole (DAPI) of hippocampal neurons allowed us to observe the nuclear morphology, as this compound is a fluorescent dye for nucleic acids. As can be seen in the images (Figure S1; A-2, B-2, C-2 & D-2), the nuclei appear rounded and clearly stained. We did not observe fragmented or lobular nuclei, typically apoptotic, nor did we observe any remarkable differences comparing the hippocampal histological sections of the control transgenic line TgAPP with respect to the WT sections, nor did we observe any differences between the quercetin and rutin treatments with respect to the control TgAPP mice.

S2. EXPRESSION OF IONOTROPIC GLUTAMATE RECEPTORS IN 45-WEEK-OLD TgAPP MICE TREATED WITH QUERCETIN AND RUTIN

For the determination of receptor density, coronal slices were chosen at the Bregma -2.54 mm position, with the intention of observing different cortical divisions as well as the hippocampus. Quantification of NMDA and AMPA receptor expression was carried out in occipital, parietal and entorhinal cortex, and at the dorsal and ventral hippocampal level. In the case of the kainate receptor, which is mainly expressed at the level of the cortex, only the occipital, parietal and entorhinal cortex were determined (Figure S2).

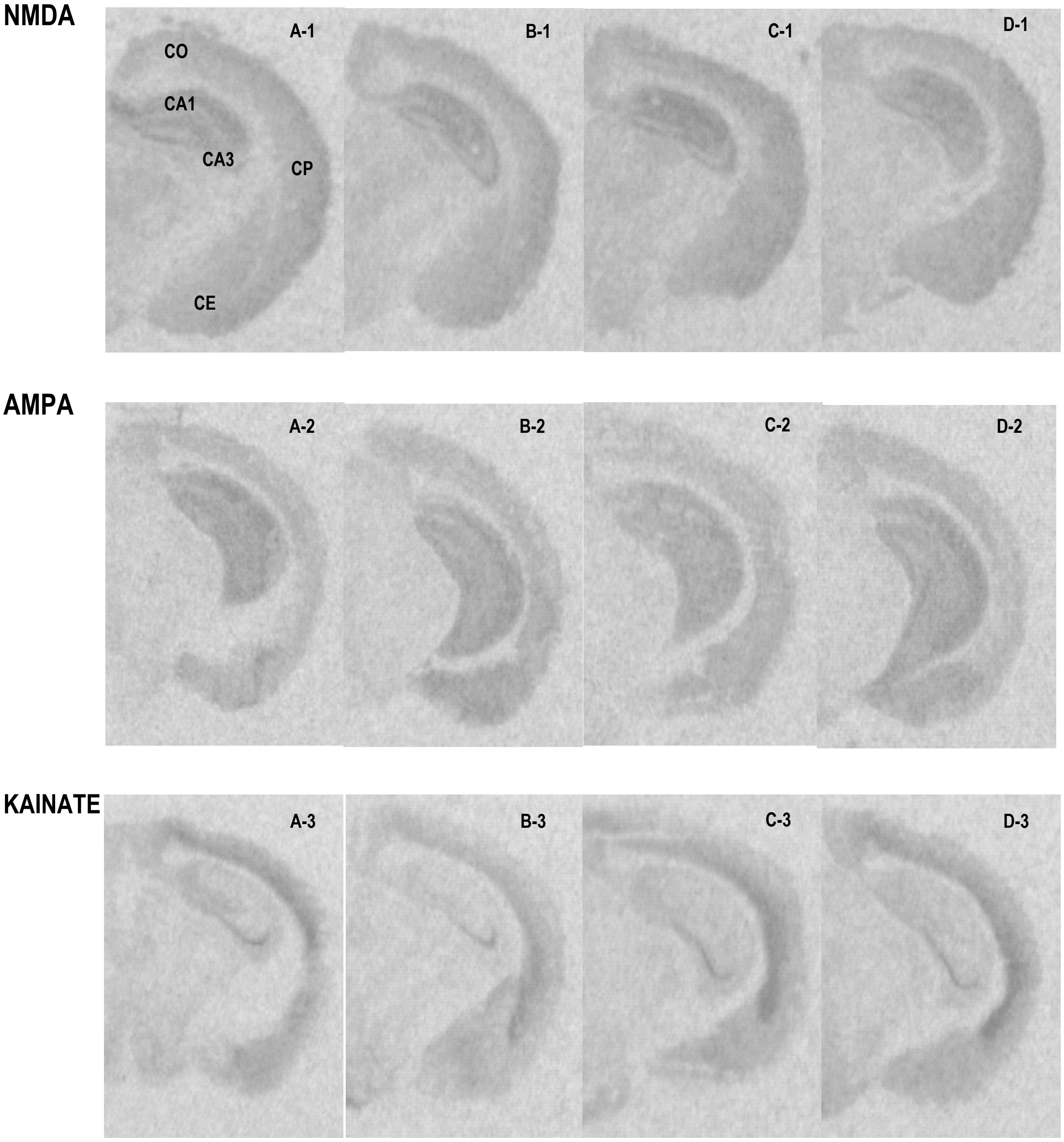

Figure S2. Representative autoradiograms of the coronal section of the left cerebral hemisphere: **A)** WT; **B)** TgAPP control; **C)** TgAPP + Quercetin; **D)** TgAPP + Rutin. No significant changes were observed between WT and TgAPP, nor between treatments. Sections were taken at the level of Bregma -2.54. (OC) occipital cortex; (PC) parietal cortex; (EC) entorhinal cortex; (CA1 and CA3) dorsal hippocampus.

Quantification of ionotropic glutamate receptor levels is presented in Table S5.

Table S5. Effect of quercetin and rutin treatment on NMDA, AMPA and kainate receptor density in occipital, parietal and entorhinal cortex and at the dorsal hippocampal level.

NMDA	WT	TgAPP	TgAPP + Quercetin	TgAPP + Rutin
**Occipital cortex**	99.463 ± 0.234	100.000 ± 0.329	102.182 ± 0.393	100.083 ± 0.538
**Parietal**	101.143 ± 0.474	99.911 ± 0.342	103.363 ± 0.650	100.708 ± 0.470
**Entorhinal**	101.007 ± 0.474	100.460 ± 0.556	103.261 ± 0.594	100.573 ± 0.469
**Dorsal Hippocampus**	99.962 ± 0.655	99.883 ± 0.467	103.762 ± 0.667	98.597 ± 1.003
**Ventral**	103.257 ± 0.624	100.000 ± 0.428	103.850 ± 0.929	102.877 ± 0.738
**AMPA**				
**Occipital cortex**	99.244 ± 0.519	100.000 ± 0.419	97.016 ± 0.641	99.685 ± 0.311
**Parietal**	99.307 ± 0.867	100.000 ± 0.873	98.222 ± 1.109	99.587 ± 0.888
**Entorhinal**	100.458 ± 1.029	100.000 ± 0.665	101.217 ± 0.653	100.286 ± 0.616
**Dorsal hippocampus**	98.664 ± 0.669	100.000 ± 0.804	97.297 ± 1.235	100.907 ± 0.804
**Ventral**	100.691 ± 0.520	100.000 ± 0.572	98.326 ± 1.148	100.380 ± 0.570
**Kainate**				
**Occipital cortex**	99.463 ± 0.234	100.000 ± 0.329	102.182 ± 0.393	100.083 ± 0.538
**Parietal**	102.812 ± 0.685	100.000 ± 0.930	99.442 ± 1.077	100.018 ± 0.865
**Entorhinal**	103.333 ± 0.769	100.000 ± 0.459	99.220 ± 0.756	104.058 ± 1.162

WT (n=3), TgAPP control (n=3), TgAPP + Quercetin (n=2) and TgAPP + Rutin (n=2). Values represent mean percentages relative to TgAPP mice ± SEM.

The values were expressed as percentages based on the values obtained for the control TgAPP mice. No significant differences were found, comparing the values obtained for the control TgAPP mice with those obtained for the WT mice. Possibly in this transgenic line the overexpression of APPswe does not involve alterations at the level of NMDA, AMPA or kainate expression. There were also no notable effects on the expression of these ionotropic receptors in the presence of quercetin or rutin treatment.
